# A meta-analysis of the effects of transcranial direct current stimulation combined with cognitive training on working memory in healthy older adults

**DOI:** 10.3389/fnagi.2024.1454755

**Published:** 2024-09-23

**Authors:** Yanxin Lv, Shuo Wu, Michael A. Nitsche, Tian Yue, Volker R. Zschorlich, Fengxue Qi

**Affiliations:** ^1^Sports, Exercise, and Brain Sciences Laboratory, Sports Coaching College, Beijing Sport University, Beijing, China; ^2^Faculty of Rehabilitation Medicine, Shandong University of Traditional Chinese Medicine, Jinan, China; ^3^Department of Psychology and Neurosciences, Leibniz Research Centre for Working Environment and Human Factors, Dortmund, Germany; ^4^University Clinic of Psychiatry and Psychotherapy, Protestant Hospital of Bethel Foundation, University Hospital OWL, Bielefeld University, Bielefeld, Germany; ^5^German Center for Mental Health (DZPG), Bochum, Germany; ^6^Faculty of Philosophy, Institute of Sports Science, University of Rostock, Rostock, Germany; ^7^Faculty of Interdisciplinary Research, Department of Ageing of Individuals and Society, University of Rostock, Rostock, Germany; ^8^Department of Sport Science, University of Oldenburg, Oldenburg, Germany

**Keywords:** transcranial direct current stimulation, older adult, working memory, cognitive training, cognitive function

## Abstract

**Background:**

Working memory (WM) loss, which can lead to a loss of independence, and declines in the quality of life of older adults, is becoming an increasingly prominent issue affecting the ageing population. Transcranial direct current stimulation (tDCS), a non-invasive brain stimulation technique, is emerging as a potential alternative to pharmacological treatments that shows promise for enhancing WM capacity and May enhance the effects of cognitive training (CT) interventions.

**Objective:**

The purpose of this meta-analysis was to explore how different tDCS protocols in combination with CT enhanced WM in healthy older adults.

**Methods:**

Randomized controlled trials (RCTs) exploring the effects of tDCS combined with CT on WM in healthy older adults were retrieved from the Web of Science, PubMed, Embase, Scopus and the Cochrane Library databases. The search time period ranged from database inception to January 15, 2024. Methodological quality of the trials was assessed using the risk-of-bias criteria for RCTs from the Cochrane Collaboration Network, and RevMan 5.3 (Cochrane, London, United Kingdom) was used for the meta-analysis of the final literature outcomes.

**Results:**

Six RCTs with a total of 323 participants were ultimately included. The results of the meta-analysis show that tDCS combined with CT statistically significantly improves WM performance compared to the control sham stimulation group in healthy older adults [standard mean difference (SMD) = 0.35, 95% CI: 0.11–0.59, *I*^2^ = 0%, *Z* = 2.86, *p* = 0.004]. The first subgroup analysis indicated that, when the stimulus intensity was 2 mA, a statistically significant improvement in WM performance in healthy older adults was achieved (SMD = 0.39, 95% CI: 0.08–0.70, *I*^2^ = 6%, *Z* = 2.46, *p* = 0.01). The second subgroup analysis showed that long-term intervention (≥ 10 sessions) with tDCS combined with CT statistically significantly improved WM compared to the control group in healthy older adults (SMD = 0.72, 95% CI: 0.22–1.21, *I*^2^ = 0%, *Z* = 2.85, *p* = 0.004).

**Conclusion:**

tDCS combined with CT statistically significantly improves WM in healthy older adults. For the stimulus parameters, long-term interventions (≥ 10 sessions) with a stimulation intensity of 2 mA are the most effective.

## 1 Introduction

From 2020 to 2030, the number of people aged >60 years is expected to increase from 1 billion to 1.4 billion, according to statistics provided by the World Health Organization. By 2050, the global population aged >60 years will double to 2.1 billion people (Sanderson et al., 2017). In other words, the centre of gravity of the global population distribution is shifting toward older population groups, a phenomenon known as population ageing. With this ageing population growth, more older adults than ever are facing a decline in a range of cognitive functions, including working memory (WM) and attention (Grover et al., 2023; Assecondi et al., 2022; Antonenko et al., 2022; Antonenko et al., 2018; Antonenko et al., 2023; Aksu et al., 2023). It has been well documented that some cognitive abilities decline with age, even among healthy older adults without neurological disorders or dementia (Woods et al., 2018), leading to impairments in various cognitive domains, including attention, language, and WM (Hyer et al., 2015). WM decline is particularly prevalent in this population. WM performance involves frontal lobe, hippocampal and temporal lobe structures, with a specific importance of the prefrontal cortex (PFC) (D'esposito and Postle, 2015), which regulates a variety of executive functions required for higher-level cognitive task performance.

Working memory requires temporary storage and online manipulation and control of information (Brunoni and Vanderhasselt, 2014). Over the past few decades, the concept of WM has become well known and has been increasingly emphasized and widely used. It has been described as the cognitive center of human beings, and it is one of the most active research areas in cognitive psychology and cognitive neuroscience at present (Baddeley, 2010; Baddeley, 2000). In fact, WM also refers to a fundamental short-term cognitive process, but it emphasizes the connection between short-term memory and the work that the current person is engaged in (Baddeley, 2010). As people age, memory tends to decline. Some studies suggest that regular cognitive training (CT) can slow the decline of WM, but not all research supports this finding (Hyer et al., 2015). WM training has been proposed as an important cognitive training intervention for older adults that May benefit not only WM, but also other cognitive processes associated with it (Teixeira-Santos et al., 2022; Thams et al., 2020). Common WM tasks include n-back, digit span, and letter-spanning tasks (Nissim, 2019).

In older adults, this decline in memory capacity can have a significant negative impact on activities of daily living – for example, knowing the time to take medications, paying bills, traveling out of the house versus staying home, or completing daily errands. Such age-related cognitive deficits, driven by declines in WM, have a profound impact on activities of daily living and quality of life in older adults (Hsu et al., 2015). Thus, there is an urgent need for effective interventions to stop or slow this cognitive decline. One intervention proposed to reduce cognitive decline is CT. CT includes a set of psychological methods that involve behavioral interventions that help to build new neural networks designed to protect brain function from age-related decline (Joubert and Chainay, 2018). CT interventions can be delivered in paper-and-pencil, and computerized versions (Clare and Woods, 2008). It includes training of specific cognitive functions like WM and attention, and also is intended to trigger long-term cognitive effects that May slow the decline of WM and help maintain independence in daily life (Nissim, 2019). These interventions May activate pre-existing cognitive reserves and promote neuroplasticity in various regions of the brain, including the frontoparietal network and the hippocampus—key regions for learning, and memory formation (Raimo et al., 2023; Elmasry et al., 2015)—which are likely improving WM in older adults. It has been shown that CT May improves cognitive performance in healthy older adults (Raimo et al., 2023), and a systematic review assessing MCI the therapeutic benefits of CT in randomized clinical trials (RCTs) came to similar conclusions (Antonenko et al., 2018). These findings provide evidence for the feasibility and usability of CT.

While some forms of CT have been shown to be promising for WM improvement in healthy older adults, CT is most frequently used as an adjunctive treatment for psychological deficits in people with cognitive decline (Woods et al., 2018). There are also other methods, such as Transcranial direct current stimulation, that can help improve WM (Assecondi et al., 2022; Brunoni and Vanderhasselt, 2014; Teixeira-Santos, et al., 2022). Transcranial direct current stimulation (tDCS) is a non-invasive brain stimulation technique, that delivers a constant current to targeted areas of the brain through surface electrodes applied to the scalp (Polanía et al., 2018). Relevant mechanisms of action include an enhancement of cortical excitability by subthreshold neuronal membrane depolarization via anodal tDCS, while cathodal tDCS results in reduced excitability via neuronal membrane hyperpolarization with conventional protocols (Nitsche and Paulus, 2001). With prolonged stimulation protocols, anodal tDCS induces long-term potentiation (LTP) -like plasticity, while cathodal tDCS induces long-term depression (LTD) -like plasticity (Gonzalez et al., 2021). Mechanistically, prolonged anodal tDCS enhances the activity of n-methyl-d-aspartate (NMDA) receptors, increases glutamate concentration, and decreases *γ*-aminobutyric acid (GABA) concentration (Nitsche and Paulus, 2001; Park et al., 2014). Because of its role in altering excitability and inducing plasticity, tDCS has been shown to improve a variety of cognitive processes, including executive function, and is therefore a promising tool for enhancing the effects of CT. For cognitive decline or mild cognitive impairment (MCI), tDCS has also been shown to improve cognitive abilities (Teixeira-Santos et al., 2022; Prehn and Flöel, 2015; Cheng et al., 2015; Thams et al., 2020; Stephens and Berryhill, 2016; Šimko et al., 2021; Antonenko et al., 2019). And a large number of studies of cognitive improvement with tDCS have been conducted in experimental research and clinical settings (Antonenko et al., 2019; Brooks et al., 2021; Figeys et al., 2022).

Since CT and tDCS share cognitive facilitation, it is conceivable that both interventions May have a synergistic effect on WM in healthy older adults, improving WM, when applied together (Krebs et al., 2021). One study found that tDCS combined with CT led to significant improvements of cognitive performance in older adults with dementia (Byeon, 2019). Moreover, in a randomized double-blind crossover trial that explored the cognitive–behavioral aftereffects of tDCS combined with CT in healthy older adults, the results showed that, compared to sham stimulation, anodal tDCS improved performance accuracy of WM training (Šimko et al., 2021). Previous meta-analyses, such as the one by Indahlastari et al. (2021), have explored the efficacy of tDCS interventions in WM and other cognitive domains. However, our study further examines the specific combination of tDCS and CT in improving WM in older adults.

## 2 Methods

A systematic review and meta-analysis was performed according to the recommendations of the Cochrane group (Higgins et al., 2011), which included a literature search, screening of eligible articles according to inclusion and exclusion criteria, data extraction of outcome indicators and other relevant variables for the included articles, assessment of the quality of the risk and analysis of the results, as described below. This review and meta-analysis was conducted according to Preferred Reporting Items for Systematic Reviews and Meta-analyses guidelines (Higgins et al., 2011; Jayawant et al., 2011).

### 2.1 Literature search

We systematically searched for respective studies in PubMed, the Web of Science, Cochrane Library, Scopus and Embase databases. These databases were chosen due to their comprehensive coverage and relevance to our research topic, encompassing a wide range of biomedical, clinical, and scientific literature. The search time period was from database inception to January 15, 2024. Keywords used for the literature search included (‘transcranial direct current stimulation’ OR ‘anodal stimulation tDCS’ OR ‘tDCS’ OR ‘electric stimulation’ OR ‘non-invasive brain stimulation’ OR ‘transcranial magnetic stimulation’ OR ‘stimulation tDCS, anodal’) AND (‘cognitive training’ OR ‘training, cognitive’ OR ‘brain training’ OR ‘training, brain’ OR ‘cognitive rehabilitation’ OR ‘memory training’ OR ‘rehabilitation, cognitive’ OR ‘training, memory’) AND (‘older’ OR ‘elderly, frail’ OR ‘frail elderly’ OR ‘frail older adults’ OR ‘adults, frail older’ OR ‘frail older adult’ OR ‘older adult, frail’ OR ‘older adult’ OR ‘older adults’ OR ‘elders, frail’). These keywords were selected to capture a broad range of relevant studies involving tDCS and CT in older adults. Additionally, we explored other relevant references in the retrieved articles to ensure a comprehensive review of the literature.

### 2.2 Inclusion and exclusion criteria

Initial screening was performed by two researchers, who first read the titles and abstracts of the selected studies and then read the full texts to exclude literature that did not meet the inclusion criteria in order to determine the final inclusion status of all studies. When the two researchers did not agree, a third researcher will need to discuss the inclusion of this literature with the research. Studies were required to follow the PICOS principles for inclusion (P: population, I: intervention, C: comparator, O: outcome, S: study design), as follows: (1) participants needed to be healthy older adults >60 years old with a cognitive function score (Mini-mental State Examination/Montreal Cognitive Assessment) score of >22 points; (2) the intervention approach was CT combined with tDCS; (3) passive controls (sham stimulation) were involved; (4) outcome indicators included WM evaluations; (5) a RCT experimental design was applied; and (6) articles were published in English language. Exclusion criteria were (1) non-English language literature; (2) duplicate literature; (3) literature for which full texts were not available; (4) literature from which relevant data could not be extracted; (5) non-RCTs; and (6) literature in which trial endpoint metrics were not relevant to WM.

### 2.3 Data extraction

The following data were extracted from the included studies: (1) authors and year of publication; (2) characteristics of subjects (mean values of age, years of education, basic cognitive health indicators and their respective standard deviation values); (3) intervention parameters (frequency, intensity, duration and stimulation electrode position); (4) CT characteristics; and (5) outcome indicators (results before and after the intervention).

### 2.4 Evaluation of literature quality

The Cochrane Risk-of-Bias Assessment Tool was used to evaluate the quality of the included eligible literature. The level of risk of bias was categorized as either low risk, high risk or unknown risk of bias (Higgins et al., 2011). The risk of bias was evaluated according to the following six aspects: selection bias (random sequence generation, allocation concealment), measurement bias, follow-up bias, reporting bias, implementation bias and other biases (Yue et al., 2022). The risk-of-bias plot visualises the reliability of the included studies, showing how many studies were rated as having a low, unclear or high risk of bias (Huo et al., 2021).

### 2.5 Statistical analysis

Studies were collated and analyzed according to the tDCS and CT intervention, the number of tDCS interventions and the protocols employed, along with subgroup analyses. The outcome indicator was the change in the value of the WM task (n-back or digit span).

RevMan 5.3 (Cochrane, London, United Kingdom) was used to statistically analyze the outcome indicators of the included literature. The indicators included in the selected literature are continuous variables, so the weighted mean difference (WMD) or standardized mean difference (SMD) was used. As an indicator of the effect scale, WMD was used if the numerical variables were obtained from the same measurement method and SMD was used for comparisons including different tasks (Green et al., n.d.). The heterogeneity of studies was examined by quantitative evaluations via Cochrane’s Q and *I*^2^. *I*^2^ < 25% indicated insignificant heterogeneity, 25% < *I*^2^ < 50% indicated low heterogeneity, 50% < *I*^2^ < 75% indicated moderate heterogeneity, and *I*^2^ > 75% indicated high heterogeneity (Yue et al., 2022). When *p* > 0.1 and *I*^2^ < 50%, it was concluded that there was little heterogeneity between studies and these were analyzed by fixed-effects modeling. For larger heterogeneity between studies, random-effects modeling was applied (Yue et al., 2022). Sensitivity analyses were performed using a study-by-study approach to exclude individual study data, and publication bias was assessed by direct observation through funnel plots. *p* < 0.05 was determined as significance threshold with the exception of subgroup analyses, where it was set to *p* < 0.1 (Richardson et al., 2019).

## 3 Results

### 3.1 Literature retrieval

**Figure 1:**
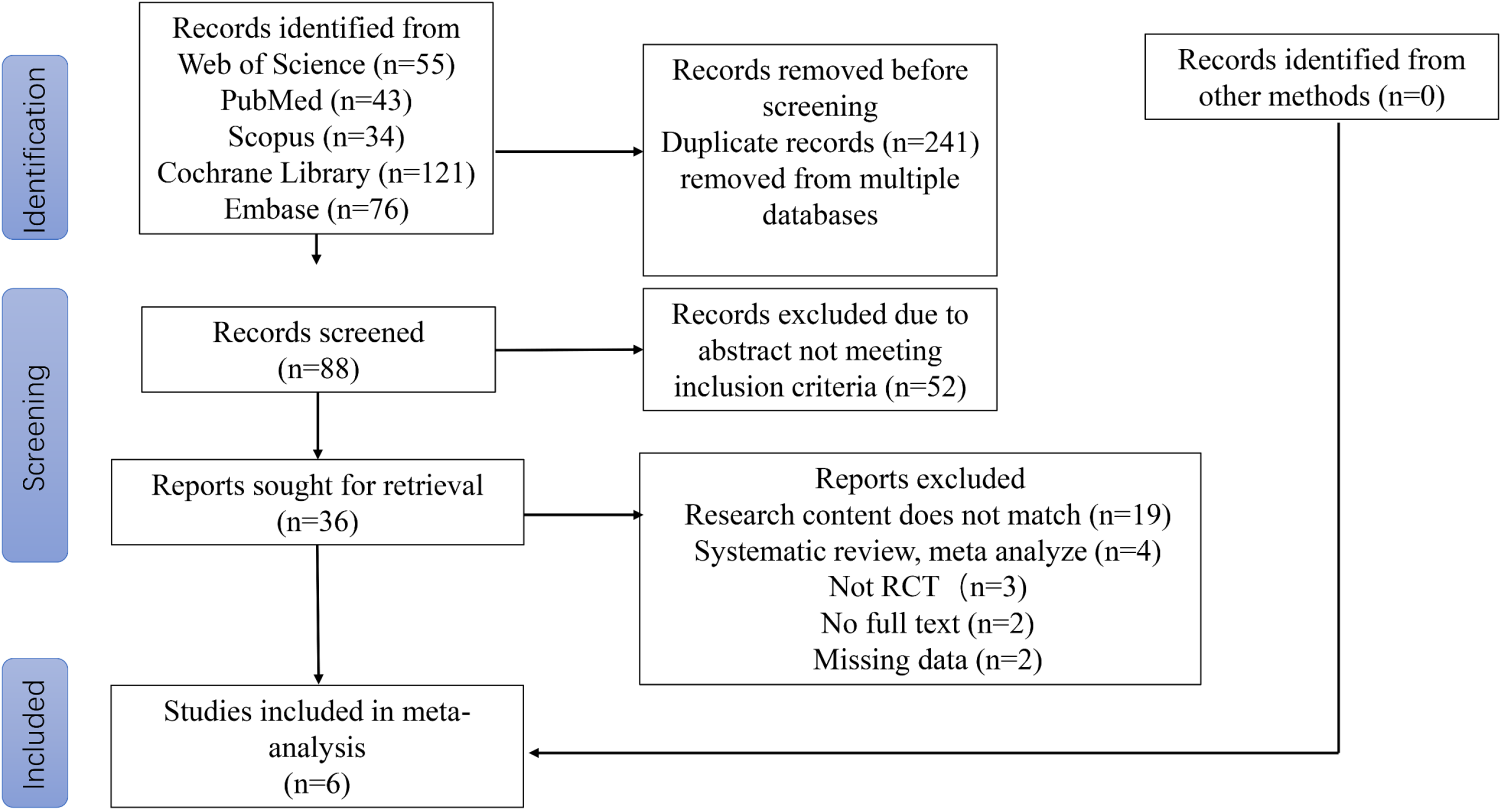
PRISMA flow diagram of literature search strategies.

The literature search and screening process is shown in Figure 1. Initial search results (*n* = 329 articles) were obtained from the Web of Science, PubMed, Scopus, EBSCO, the Cochrane Library and Embase, and duplicate literature (*n* = 241) was subsequently excluded. The remaining studies (*n* = 88) were screened, and 52 papers were additionally excluded as they did not meet the inclusion criteria. The remaining 36 articles were read in their entirety. After excluding studies that did not meet the inclusion criteria of this study (*n* = 19), were part of a systematic review or meta-analysis (*n* = 4), were not RCTs (*n* = 3), had no full-text version available (*n* = 2) or had incomplete data (*n* = 2), six articles were ultimately included in the analysis of this study.

### 3.2 Study characteristics

For the included studies, the following data are listed in Table 1: author and year of publication, number of participants, age and duration of education, basic cognitive scores, number of interventions, stimulation electrode positions, and primary outcome indicators. In the selected six studies not only tests of WM performance, but also tests of other cognitive functions were carried out; however, in the present study, only the WM task metrics were considered. Two of the publications applied a digit span task for WM performance measures (Teixeira-Santos et al., 2022; Stephens and Berryhill, 2016), while the remaining four publications used the n-back test task (Park et al., 2014; Antonenko et al., 2022; Jones et al., 2015; Perceval et al., 2020). In all six studies, the interventions were tDCS combined with CT. The most frequently stimulated brain area was the DLPFC at an intensity of 2 mA in four papers (Teixeira-Santos et al., 2022; Park et al., 2014; Stephens and Berryhill, 2016; Hausman et al., 2023) and 1 mA in two papers (Stephens and Berryhill, 2016; Antonenko et al., 2022; Perceval et al., 2020). The number of interventions ranged from 5 to 10, and the stimulation duration ranged from 10 to 30 min. Characteristics of the included studies are given in Table 1.

**Table 1:** Descriptive characteristics of the included studies.

Study	Teixeira-Santos et al. (2022)	Stephens and Berryhill (2016)	Park et al. (2014)	Antonenko et al. (2022)	Perceval et al. (2020)	Nissim (2019), Nissim et al. (2019)
Number of participants (stim/sham)	^*^54 (18/18/18)	^*^90 (30/30/30)	40 (20/20)	24/27	30/30	14/14
Mean age (stim/sham)	67.61/68.67/68.33	68.6/68.6/69.9	70.1/69.4	69.7/69.9	66.7/67.4	73.57/73.78
Mean MMSE/MoCA (stim/sham)	Mo, 22.5/23/22.56	MM, > 22	MM, 29.3/28.8	MM, 29.4/29.3	MM, 29.67/29.47	Mo, 27.85/27
stimulation tool and protocol	atDCS, 2 mA, 20 min	tDCS, 2 mA 15 min	tDCS, 2 mA, 30 min	tDCS, 1 mA, 20 min	tDCS, 1 mA, 20 min	tDCS, 2 mA, 20 min
Stimulation electrode position and size	A: L DLPFC, C: R supraorbital area, 5 × 7 cm^2^	R DLPFC; 5 × 7 cm^2^	B DLPFC; 5 × 5 cm^2^	A: L DLPFC, C: R supraorbital area, 5 × 5 cm^2^	A: L IFG; C: R supraorbital area, 5 × 7 cm^2^	A: R DLPFC; C: L DLPFC, 5 × 7 cm^2^
Number of interventions*	Once a day for 5 consecutive days	Once a day for 5 consecutive days	Twice a week for 5 weeks for a total of 10 times	9 tDCS	Once a day for 5 consecutive days	10 tDCS
Follow-up duration	15 days	1 month	7 days 28 days	1 month 7 months	1 week 3 months	2 weeks
Main outcome measures of cognitive function	Digit span	Letter span	2-back/digit span	2-back	2-back	2-back/0-back
Type of combination of CT and tDCS	Synchronous	Synchronous	Synchronous	Synchronous	Synchronous	Synchronous
CT characteristics	Dual n-back or placebo task	WM training tasks	CACT program	Lu task followed by Mdm task	explicit learning paradigm	40 min of CT per session, 20 min prior to task in conjunction with tDCS
Double-blind method	Double-blind	Single-blind	Double-blind	Single-blind	Double-blind	Triple-blind

Stim, stimulation; atDCS, anodal tDCS; stDCS, sham tDCS; L DLPFC, left dorsolateral prefrontal cortex; R DLPFC, right dorsolateral prefrontal cortex; B DLPFC, bilateral dorsolateral prefrontal cortex; A, anode; C, cathode; MMSE, MM, Mini-mental state examination; MoCA, Mo, Montreal Cognitive Assessment; IFG, Inferior Frontal Gyrus; CACT: computer-assisted cognitive training; Lu: Letter updating; Mdm, Markov decision making; *54 (30/30/30), atDCS + CT, stDCS + CT, double-sham; *90 (30/30/30), sham, 1 mA tDCS, 2 mA tDCS.

### 3.3 Quality assessment

**Figure 2:**
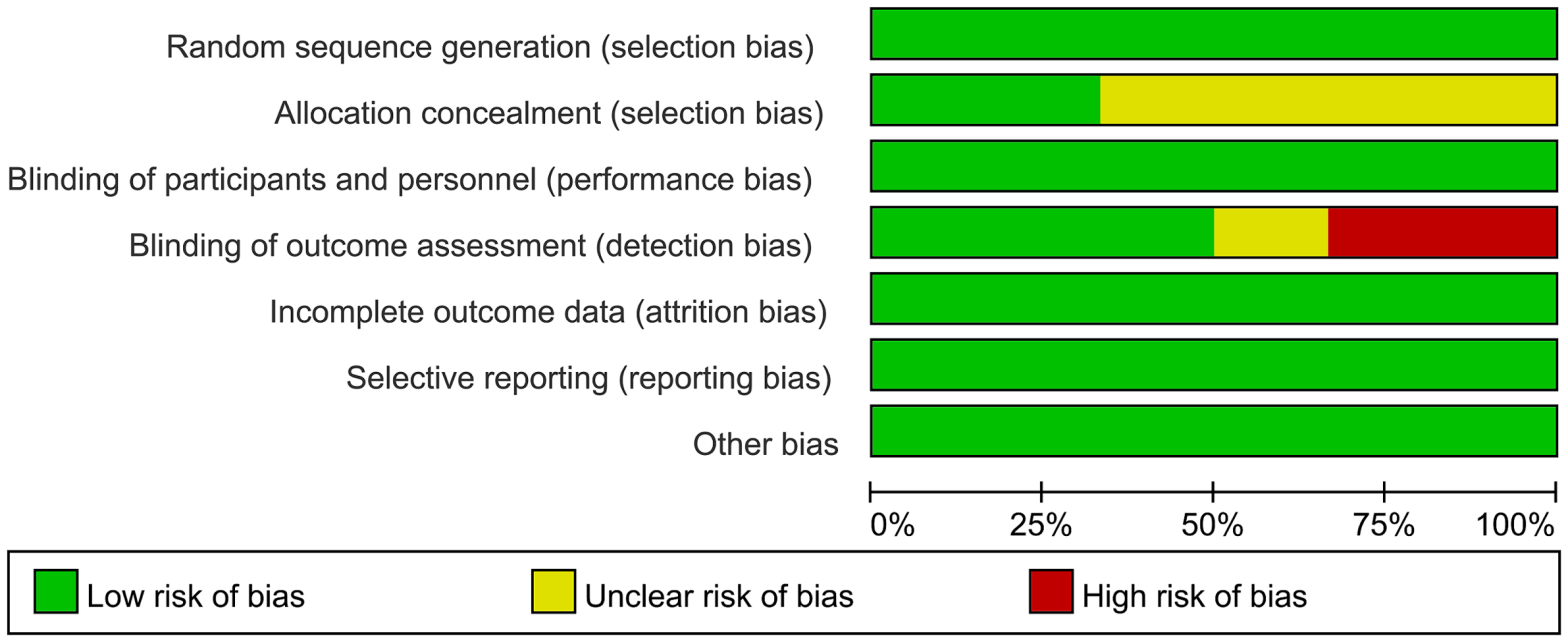
Summary of risk of bias by domain.

Two researchers independently assessed the quality of the included studies and resolved discrepancies through discussion. The quality of the included studies was assessed via a Cochrane Collaboration tool (Higgins et al., 2011), and, as shown in the risk-of-bias graph, low risk dominated in the domains of selection bias, performance bias, detection bias and reporting bias. Allocation concealment was not reported in most of the included studies. For blinding, triple-blinding was reported in only one study (Hausman et al., 2023). Two studies used single blindness and three studies used double blindness. The overall quality of the included studies was relatively good, as shown in Figure 2.

### 3.4 Sensitivity analysis

In order to verify the reliability of the results, we excluded the six studies one by one and checked for each exclusion if it had a significant effect on the pooled results. Sensitivity analysis showed that no exclusion of a single study relevantly affected the results of this meta-analysis, implying that the current analysis results had good stability.

### 3.5 Meta-analysis results

#### 3.5.1 Effects of tDCS combined with CT on WM

**Figure 3:**
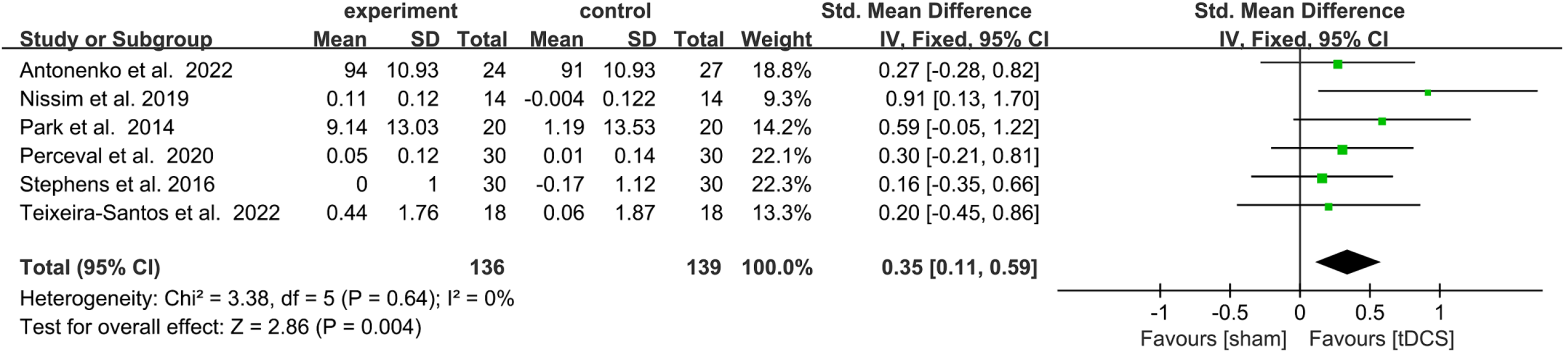
Forest plot depicting the effect of CT combined with actDCS vs. stDCS on working memory in healthy older adults. actDCS, active transcranial direct current stimulation; stDCS, sham transcranial direct current stimulation.

Due to the low heterogeneity of this meta-analysis (*p* > 0.5, *I*^2^ < 50%), it was performed using a fixed model. SMD was used as the effect scale indicator because of the different measurement units of each data set. The results show that active tDCS combined with CT statistically effectively improved WM in healthy older adults compared to the control protocol [SMD = 0.35, 95% confidence interval (CI) = 0.11–0.59, *I*^2^ = 0%, *Z* = 2.86, *p* = 0.004, Figure 3].

#### 3.5.2 Effects of stimulus intensity on WM

**Figure 4:**
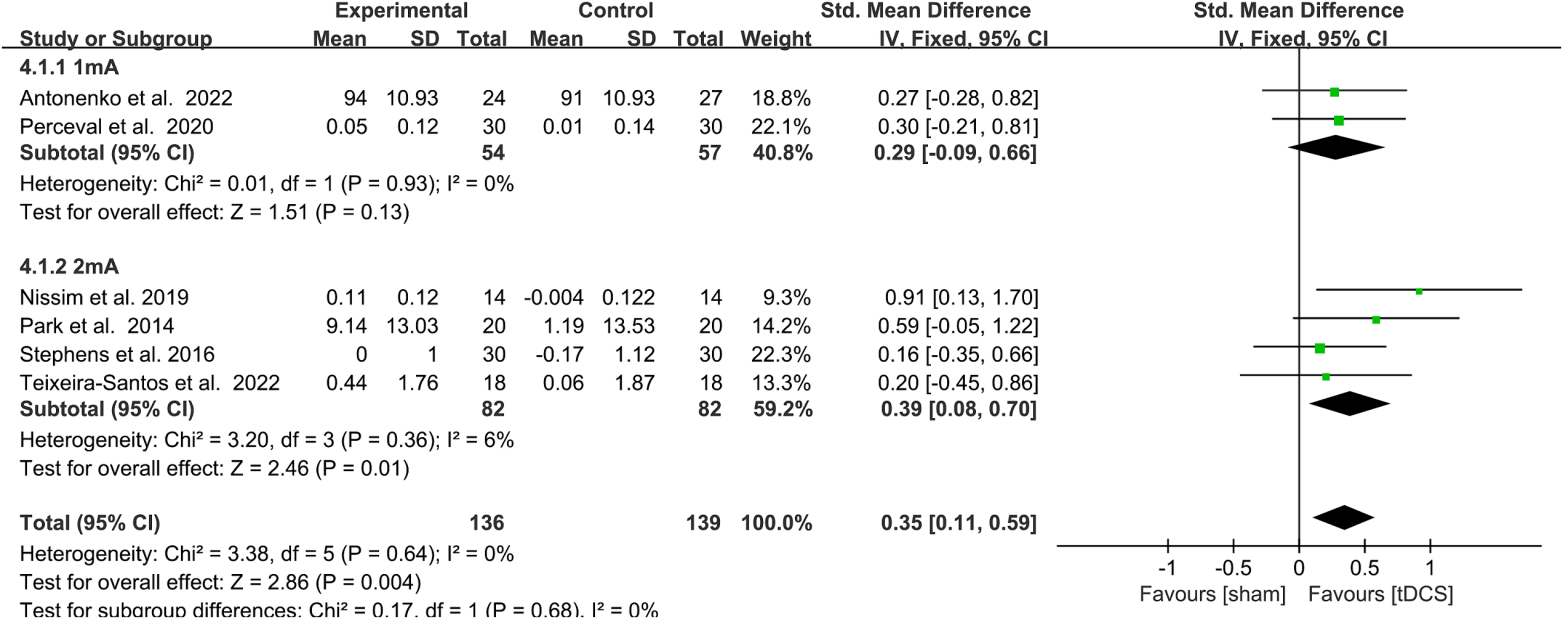
Forest plot of subgroup analysis by a different model of stimulus intensities (1 mA, 2 mA). tDCS, transcranial direct current stimulation.

A subgroup analysis of the WM performance of healthy older adults was performed according to the stimulus intensity (1 vs. Two mA). Heterogeneity was low after subgroup and fixed model analyses, so fixed model analysis was used. When the stimulus intensity was 2 mA, it statistically significantly improved WM in healthy older adults (SMD = 0.39, 95% CI = 0.08–0.70, *I*^2^ = 6%, Z = 2.46, *p* = 0.01), while no significant performance improvement emerged with a stimulus intensity of 1 mA. There was no statistically significant subgroup differences [Chi^2^ = 0.17, *p* = 0.68 (> 0.1), Figure 4].

#### 3.5.3 Effects of different numbers of interventions on WM

**Figure 5:**
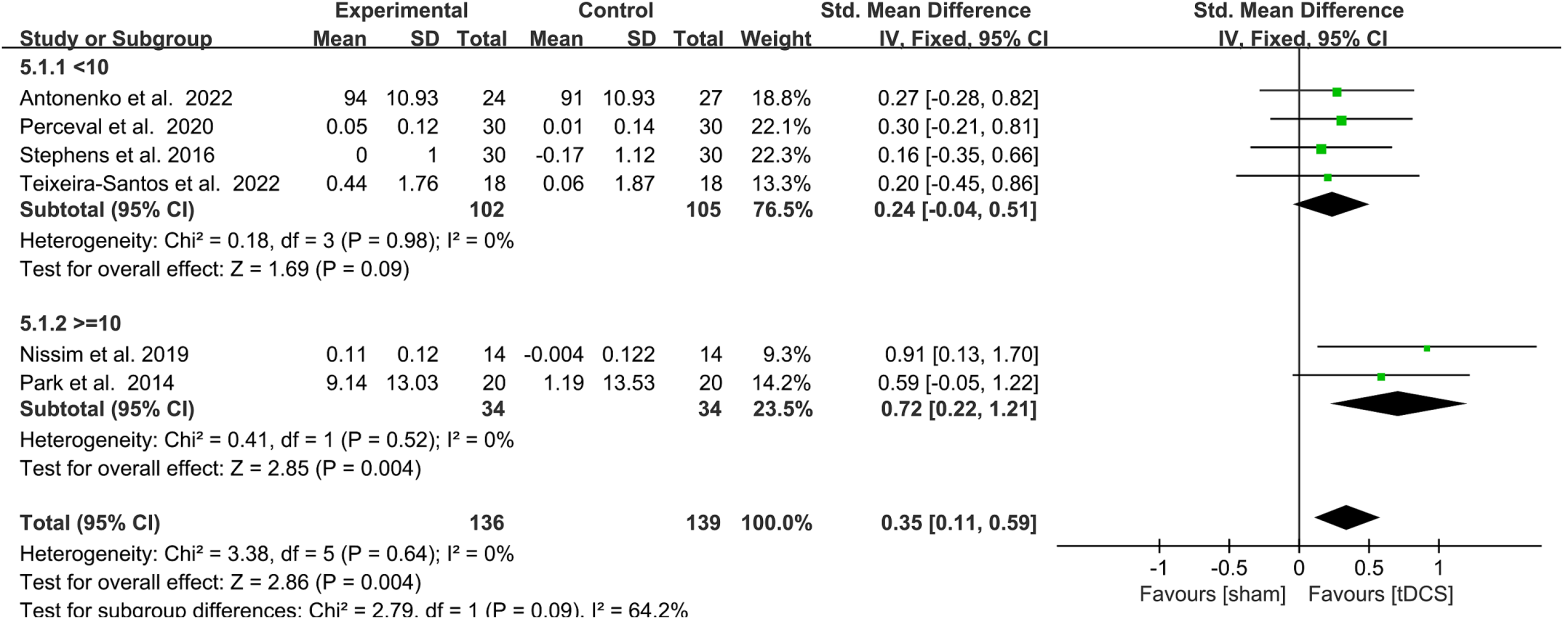
Forest plot of subgroup analysis by a different model of number of interventions (< 10 times, > = 10 times). tDCS, transcranial direct current stimulation.

A total of 6 trails was included in this subgroup analysis, of which 4 studies had <10 interventions, and 2 studies had > = 10 interventions. A subgroup analysis of the WM capacity of healthy older adults was performed according to the number of interventions (< 10 vs. ≥ 10 sessions). Heterogeneity was low (*I*^2^ = 0) after subgroup analysis, and this a fixed model analysis was used. The outcome measures showed that > = 10 stimulation sessions (SMD = 0.72, 95% CI = 0.22–1.21, *I*^2^ = 0%, *Z* = 2.85, *p* = 0.004) had a statistically significant improving effect on WM performance in healthy older adults. In contrast, < 10 stimulation sessions (SMD = 0.24, 95% CI = −0.04-0.51, *I*^2^ = 0%, *Z* = 1.69, *p* = 0.09) did not significantly improve WM performance in healthy older adults. The test for subgroup differences showed a statistically significant subgroup effect [Chi^2^ = 2.79, *p* = 0.09 (< 0.1), Figure 5] (Richardson et al., 2019). This indicated that different numbers of interventions modified the effect of the real, compared with sham group.

### 3.6 Publication bias

**Figure 6:**
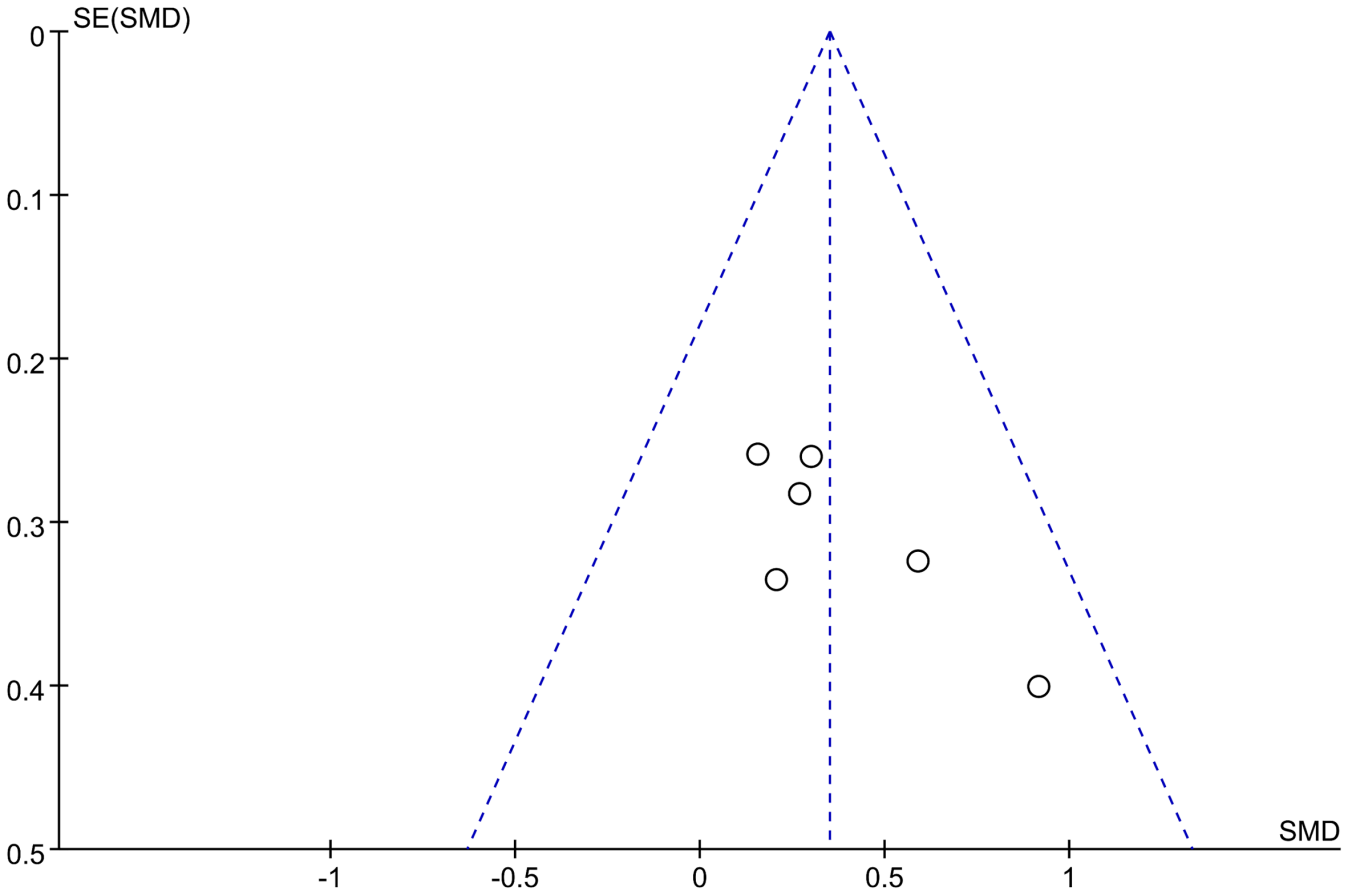
A funnel plot showing publication bias among included studies.

A possible publication bias in this study was examined by a funnel plot (Figure 6). The symmetry of the funnel plot implies the absence of a relevant bias.

## 4 Discussion

In total, six randomized sham-controlled trials with different numbers of stimulation sessions, and different stimulus parameters were included. The results of the quality assessment indicate that all of these studies were of sufficiently high quality. All studies used standard randomization and blinding methods, and described the employed methods adequately. Funnel plot analysis also showed a low risk of publication bias.

Previous meta-analyses supported the cognitive benefits of tDCS (Indahlastari et al., 2021). We extend these findings by examining the WM benefits of tDCS combined with CT in healthy older adults. This approach provides new insights into how combining tDCS with cognitive interventions may enhance cognitive outcomes in this population. The results showed that combined CT with a stimulus intensity of 2 mA and >=10 tDCS stimulations statistically significantly improved WM in healthy older adults compared to sham stimulation. This result indicates that specific tDCS parameters are critical for intervention effectiveness. The combination of 2 mA stimulation intensity and CT not only promotes new connections between nerve cells in the brain, but also triggers significant excitatory changes in the cerebral cortex, thus promoting neuroplasticity and memory consolidation (Benzing and Schmidt, 2017; Albizu et al., 2023; Antonenko et al., 2019). In addition, a greater number of stimuli (10 or more) may provide a cumulative effect, continuously enhancing brain function (Hanley et al., 2020). After each stimulation, the strengthening of neuronal connections and optimization of the neural network may take some time to consolidate with repeated stimulation, thereby achieving significant effects after multiple stimulations (Fröhlich et al., 2014; Müller et al., 2022). In our study, we found that null findings in individual studies might be attributed to the following factors: (1) effect sizes smaller than the study was powered to detect; (2) random variance; (3) the use of suboptimal stimulation parameters, such as fewer sessions and current intensities below 2 mA. These factors should be considered when interpreting null results. A meta-analysis of CT combined with non-invasive brain stimulation (NIBS) found that joint application did not produce improvements in overall cognition, although all studies reported positive effects of CT on overall cognition (Begemann et al., 2020). In the analytical discussion, it is noted that fewer studies were included in this meta-analysis. Future studies need to recruit larger samples to ensure sufficient statistical power for analyzing the effects of CT combined with tDCS. Compared to healthy older adults, in the results of Leung's meta-analysis, tDCS poles and CT had a moderately significant effect on WM in patients with Parkinson's with improved executive function and sustained improvement at follow-up after three months (Lawrence et al., 2017). Some studies have found significant improvements in cognition in older people with NIBS, with one study finding that high-frequency repetitive transcranial magnetic stimulation was more effective than tDCS combined with CT in improving overall cognition, including WM, and dementia patients may respond better to repetitive transcranial magnetic stimulation and tDCS than MCI patients (Müller et al., 2022; Liu et al., 2020). However, there are fewer studies of NIBS approaches other than tDCS and most have smaller sample sizes, so future studies should explore the effects of NIBS combined with CT on WM in healthy older adults.A large body of evidence suggests that NIBS favors cognitive improvement and maintenance, and that tDCS in particular have a greater impact on WM (Hara et al., 2021; Razza et al., 2023). However, different stimulus intensities and number of interventions can have different effects on WM improvement (Figeys et al., 2022; Arciniega et al., 2018). Subgroup analyses were conducted for this purpose in order to identify the reasons for heterogeneity between studies. In the first subgroup analysis the results showed that 1 mA tDCS combined with CT had no significant improvement in WM, whereas 2 mA tDCS combined with CT statistically significantly improved WM. A study by Reinhart and Woodman (Reinhart and Woodman, 2014) similarly found that a tDCS intensity of 2 mA was significantly better than the 1 mA group and the sham group in enhancing WM task performance. This is consistent with our meta-analysis results, supporting the effectiveness of 2 mA intensity in inducing neural excitability changes and enhancing cognitive function. In contrast, the study by Gill et al. (2015) did not find significant improvement in WM with tDCS 1 mA intensity, further emphasizing the importance of adequate intensity. Stephens (Stephens and Berryhill, 2016) showed that, while participants who received tDCS with intensities of 1 or 2 mA showed improvements in the training task later in the intervention, 2-mA tDCS led to significantly larger in tasks related to WM at the 1-month follow-up. Functional neuroimaging studies in humans have previously demonstrated that the frontal and parietal cortices are activated during the performance of WM tasks (Nissim, 2019). Enhanced frontoparietal connectivity May be a mechanism supporting WM capacity (Park et al., 2014; Edin et al., 2009). When the stimulus intensity reaches 2 mA, tDCS enhances the coherence between these areas, resulting in a more significant improvement in WM (Cheng et al., 2015; Stephens and Berryhill, 2016; Šimko et al., 2021; Assecondi et al., 2022; Brambilla et al., 2021).

We also performed subgroup analysis regarding the number of intervention sessions, studies by Fröhlich et al. (2014) also noted that longer cycles of tDCS (e.g., 10 or more sessions) had more pronounced effects in enhancing cognitive function. Their research indicates that repetitive stimulation can produce cumulative effects, gradually enhancing WM function by repeatedly strengthening neural connections. This is consistent with the results of our meta-analysis, emphasizing the importance of continuous multiple stimulation. However, short-term interventions (e.g., fewer than five sessions), such as the study by Nilsson et al. (2015), did not observe significant improvement effects, possibly due to the insufficient number of stimulations to produce a cumulative effect. Therefore, we believe that the number of interventions is an important factors for improving WM in older adults (Katz et al., 2017). If the number of stimulation interventions increases, the excitability increases in their motor cortex, which induces higher levels of stress hormones and increases the production of new neurons in the hippocampus as a way to improve learning and memory (Antonenko et al., 2019; Arciniega et al., 2018).

The current findings indicates that tDCS combined with CT offers a promising new approach to improving WM decline in healthy elderly people. Although tDCS combined with CT statistically significantly improved WM in older adults, the effect size was small (0.35). Specifically, while this improvement is statistically significant, its practical significance and impact on WM performance or quality of life May be limited. Thus, further research is needed to examine whether longer training times or different tDCS parameters May produce greater effects. Additionally, future studies should include an assessment of the clinical significance of the effect size to determine whether such small improvements are sufficient to provide substantial benefits. As the limits of safe stimulation have not be defined so far and no serious adverse effects have been reported, thus strengthening of protocols might be possible and follow-up assessment to consider long-term risks and benefits (Edin et al., 2009; Wassermann et al., 1998). In addition to uncovering safety issues, several open questions should be addressed in future research with appropriate experimental designs. Firstly, future research should further validate the long-term effects of 2 mA and more than 10 sessions of stimulation, and explore the response differences among different individuals, such as those of varying ages and cognitive levels. Second, previous studies have shown that other NIBS, such as tACS, TMS, in combination with CT can also significantly enhance the training effect (Ditye et al., 2012; Volkmann et al., 2013; Rabey and Dobronevsky, 2016), but fewer studies have been involved compared to tDCS, and thus there is a need to assess the effects of more NIBS on the aging brain when used in conjunction with CT or other behavioral interventions. Thirdly, it is important to determine the different physiological responses to brain stimulation in people of different ages so that the stimulation programme can be adjusted if necessary. There is also a need to analyze different population subgroups with long-term follow-up to identify the target populations that will benefit most from stimulation and to determine the sustainability of the beneficial effects. Finally, it is necessary to better elucidate the underlying neural mechanisms involved in the positive effects induced by stimulation.

There are some other concerns that need to be taken into account in the present study. First, there are fewer studies of tDCS combined with CT for WM in healthy older people, so the number of studies included was only 6, which May affect the accuracy of the results. Second, the reliability of the results May be affected by the fact that the older population has different ages and levels of health, and different ways of testing the outcome indicators. Thirdly, the article only includes literature in English which may result in a lack of relevant research data.

## 5 Conclusion

This systematic review and meta-analysis indicate that tDCS as an intervention has a significant effect in combination with CT on improving WM in older adults. tDCS based on the results of this analysis there are hints that an intensity of 2 mA, and interventions including more than 10 sessions are better suited than lower dosages, and number of sessions to improve and enhance WM in healthy older adults.

## 6 Implications and prospects

This meta-analytic study found that tDCS combined with CT improved WM in older adults, when specific protocols were applied. Some limitations of this analysis should however be considered. First, the included studies were methodologically heterogeneous, including stimulation protocols, participant inclusion criteria, and experimental design. Second, only a relatively minor number of studies was available for the analysis. In the future, the effects of different tDCS protocols (stimulation duration, intensity and frequency) on WM as well as cognitive performance with regard to other domains in older adults with different health conditions should be explored. Further studies need to conducted to provide a more comprehensive and accurate theoretical basis for tDCS interventions aimed to improve cognitive functions in older adults.

## Data Availability

The original contributions presented in the study are included in the article/supplementary material, further inquiries can be directed to the corresponding author/s.
